# Synthetic Cannabis-Induced Mania

**DOI:** 10.1155/2015/310930

**Published:** 2015-03-09

**Authors:** Mehmet Fatih Ustundag, Esra Ozhan Ibis, Atakan Yucel, Halil Ozcan

**Affiliations:** ^1^Department of Psychiatry, Medical Faculty, Ataturk University, 25240 Erzurum, Turkey; ^2^Department of Child and Adolescent Psychiatry, Medical Faculty, Ataturk University, 25240 Erzurum, Turkey

## Abstract

Synthetic cannabinoids (SC), cannabinoid 1 and cannabinoid 2 receptors agonists, are the psychoactive substances. SC was originally produced to treat medical conditions. Compared to other narcotics, SC is easier to obtain, cheap, and highly potent and has a long half-life. In addition, routine analysis does not detect SC, which has led to widespread use. A case is presented that manic episode was developed with the use of SC. Hospitalization and admission to psychiatric units depending on SC use have been observed mostly with psychosis. Although SC-induced affective symptoms were mentioned in the literature, mania has not been reported before. We aimed to discuss the psychiatric conditions induced by widespread use of SC via our case.

## 1. Introduction

Synthetic cannabinoids (SC), cannabinoid 1 and cannabinoid 2 receptors agonists, are the psychoactive substances [1]. The use of SC is spreading throughout all over the world, being called “bonsai” and “Jamaica” in our country, Turkey, and “chronic” and “K2” in the rest of the world. SC was originally produced to treat medical conditions until its addictive features were determined [2]. Compared to other narcotics, SC is easier to obtain, cheap, and highly potent and has a long half-life. In addition, routine analysis does not detect SC, which has led to widespread use [3].

Cannabinoids cause various physical side effects such as ST elevated myocardial infarction, respiratory difficulty, palpitations, arrhythmia, chest pain, cardiotoxicity, hypertension, acute renal failure, rhabdomyolysis, acidosis, hypokalemia, hyperthermia [3, 4], and mental side effects such as panic, anxiety, confusion, hallucinations, agitation, irritability, attention and memory distortion, paranoia, thought disorder, disorganized behavior, affective changes, and suicidal and homicidal thoughts [5]. Hospitalization and admission to psychiatric units depending on SC use have been observed mostly with psychosis. Although SC-induced affective symptoms were mentioned in the literature, mania has not been reported before [5, 6]. A case of an SC-induced manic attack is presented.

## 2. Case Report 

Our patient is an 18-year-old single boy who dropped out of high school in the second year. He lived with his brother. During the previous several months, the brother had observed increasing self-talking and laughing, looking at a point plunge for a long time, an increase in speech and spending money, a great deal of interest in religion, and a decrease in the need for sleep. In addition, the patient thought that he was an angel, demon, and prophet. He was admitted to our outpatient clinic by his relatives. In his psychiatric examination, the patient was conscious, and his orientation was completely normal. His immediate, recent, and remote memory and mental capacity were also normal. Affect was irritable, and his mood was euphoric. His movements, spontaneous speech, and thought speed had increased. Reasoning and insight were limited. Mystical and grandiose delusions compatible with his mood and increased self-esteem were observed. There were no hallucinations.

In the patient's history according to his parents, there were no psychiatric and mental problems. The patient's prenatal, natal, and postnatal period was completely normal. In childhood, he was quite calm. His school performance was good. When he was 15 years old, symptoms of conduct disorder such as school truancy, lying, being involved in a fight, coming home late, and using illegal stimulant drugs were stated. It was reported that drug use combined with volatile substances had begun 4 years before. After several months, he used cannabis powder during the following 2 years. He switched from cannabis powder to synthetic cannabis 6 months before presenting at our clinic.

The physical and neurological examination revealed no pathological symptoms. Additionally, there were no remarkable findings on the hemogram, liver function tests, renal function tests, thyroid function tests, electroencephalography, electrocardiography, chest X-ray, brain magnetic resonance imaging, screening for syphilis, HIV, HBV, HCV, and urine analysis (urine cocaine, cannabis, opioid, amphetamine, and benzodiazepine levels). According to the DSM-V, his diagnosis was compatible with substance-induced bipolar disorder. The patient was admitted to our inpatient clinic. His young mania rating scale (YMRS) was scored as 30/60. Olanzapine 10 mg/day, valproic acid 500 mg/day, quetiapine 200 mg/day, and lorazepam 0,5 mg/day were initiated. The doses were gradually increased and final doses were Olanzapine 20 mg/day, valproic acid 1500 mg/day, quetiapine 400 mg/day, and lorazepam 1 mg/day. On the 15th day of hospitalization, on the psychiatric examination, his mood was euthymic and speed of speech and amount of sleep were normal. However, mystical and grandiose delusions were ongoing. His YMRS was scored as 19/60. At the clinical follow-up, his irritability and sleeplessness were recovered. No symptoms of substance withdrawal were observed, and lorazepam was stopped. On the 30th day of hospitalization, his YMRS was scored as 6/60, and his psychiatric condition was very close to normal. In addition, a significant decrease in mystical and grandiose delusions was observed. The patient was discharged with treatment of Olanzapine 20 mg/day, valproic acid 1500 mg/day, and quetiapine 400 mg/day. His psychiatric condition was stable in the first month of follow-up with current treatment. Follow-up is ongoing.

## 3. Discussion

In recent years, production and consumption of psychoactive drugs, namely, herbal highs and legal highs, have increased with the medical objectives to be produced by SC derivatives [7]. Use of SC is now a serious health problem. SC is also being sprayed on different herbal mixtures and similar to cannabis smoking as mixtures of herbal cigarettes are released [1, 8]. SC is a more powerful stimulant than natural cannabinoids. SC is easy to obtain and undetermined in routine tests. SC is also easily accessible [9]. Due to these factors, use of SC has become more widespread. These factors were very effective for use of SC in our patient. In the previous 6 months, several times per week, our patient stated he had used SC in the form of a cigarette. The literature on prolonged use of SC is limited. However, symptoms similar to those for prolonged use of natural cannabinoids are expected. Long-term use of natural cannabis is related to psychotic symptoms, relapse of psychosis, and worsening of psychotic symptoms. Similarly, psychotic symptoms such as visual hallucinations, paranoid delusions, thought block, and disorganized speech have been reported with long-term use of SC [5, 10]. Furthermore, natural cannabis-induced symptoms of mania, hypomania, and agitations have been reported. These symptoms may be observed in SC use; however, data are lacking [11]. In our case, there was no psychiatric history without adolescent conduct disorder symptoms before SC use. Additionally, his parents had no history of psychiatric disorder. Although the patient consumed various substances and drugs, especially natural cannabis, manic symptoms were not observed. These symptoms emerged shortly after the use of SC. Therefore, the patient's mania may have been caused by SC. This is the first case of manic episode with psychotic symptoms induced by SC published in the literature. The impact of SC may be more destructive than that of other substances due to features of SC. However, this observation must be supported with further studies.
